# Anti‐TIF1γ antibody‐positive dermatomyositis associated with durvalumab administration in a patient with lung and oesophageal cancers

**DOI:** 10.1002/rcr2.736

**Published:** 2021-03-23

**Authors:** Ryosuke Imai, Sumie Ikemura, Torahiko Jinta

**Affiliations:** ^1^ Department of Pulmonary Medicine, Thoracic Center St. Luke's International Hospital Tokyo Japan; ^2^ Department of Dermatology St. Luke's International Hospital Tokyo Japan

**Keywords:** Dermatomyositis, durvalumab, immune checkpoint inhibitor, intravenous immunoglobulin

## Abstract

We report a case of anti‐transcriptional intermediary factor 1γ (TIF1γ) antibody‐positive dermatomyositis following durvalumab treatment. The patient was successfully treated with pulse steroid therapy, high‐dose intravenous immunoglobulin (IVIg), and tacrolimus. Durvalumab may induce dermatomyositis, and early diagnosis and aggressive therapy are crucial to prevent severe dermatomyositis, which is potentially treatable.

## Graphical abstract

Durvalumab can cause serious dermatomyositis. Early diagnosis and aggressive therapy are crucial to prevent severe dermatomyositis, which is potentially treatable. Continuous monitoring for muscular symptoms, creatine phosphokinase level, and skin rash in patients administered with durvalumab is vital.

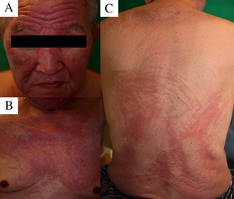

## Introduction

Durvalumab is an immune checkpoint inhibitor (ICI) that inhibits the programmed cell death ligand 1 (PD‐L1), thereby stimulating the immune system to react against cancer cells [1]. However, it is reported that ICI causes immune‐related adverse events including rash, colitis, thyroiditis, hypophysitis, hepatitis, pneumonitis, and neuromuscular disorders.

Here, we present the first report of anti‐transcriptional intermediary factor 1γ (TIF1γ) antibody‐positive dermatomyositis following durvalumab treatment, successfully treated with pulse steroid therapy, high‐dose intravenous immunoglobulin (IVIg), and tacrolimus.

## Case Report

A 74‐year‐old Japanese man, who is a former smoker with 80 pack‐year history and no history of autoimmune or neuromuscular diseases, was diagnosed with synchronous double cancers: adenocarcinoma of the left upper lobe (T1N3M0, stage IIIB, PD‐L1 expression <1%) and oesophageal cancer (T3N0M0, stage II). Concurrent chemoradiotherapy and radiotherapy (60 Gy in 30 fractions) with cisplatin (80 mg/m^2^ on day 1) and vinorelbine (20 mg/m^2^ on days 1 and 8) were started, followed by durvalumab administration (10 mg/kg on day 1). After the third dose, the patient was admitted with a three‐week history of rashes on his face and neck, and muscle weakness of the limbs.

**Figure 1:**
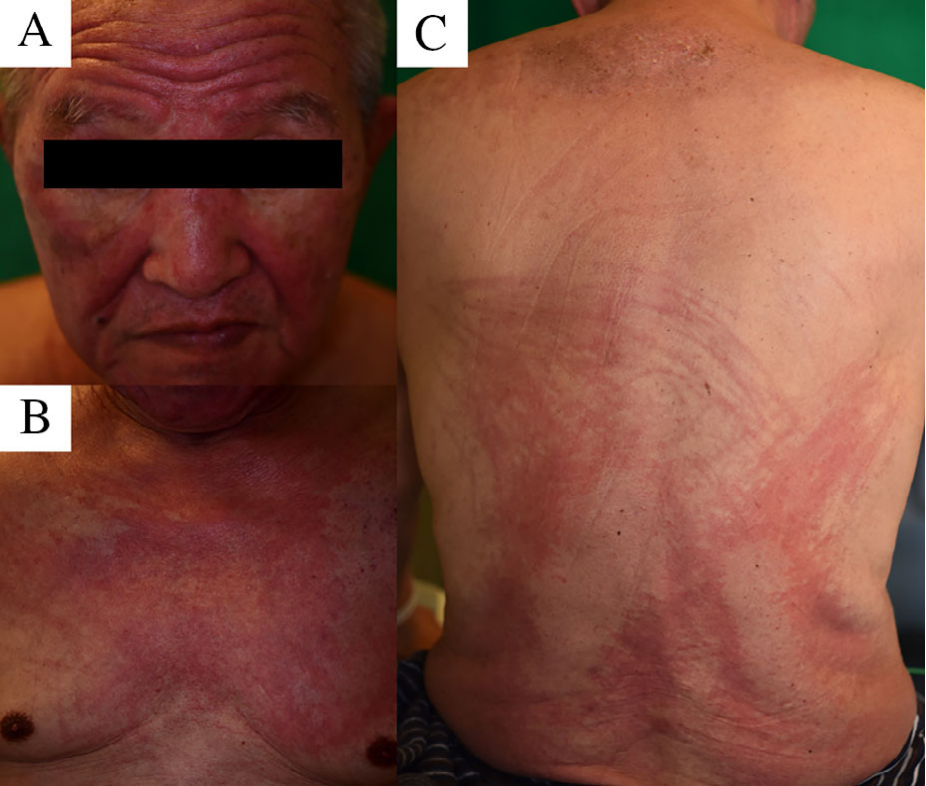
Heliotrope rash on patient's face (A), shawl sign (B), and macular violaceous erythema over his back (C).

**Figure 2:**
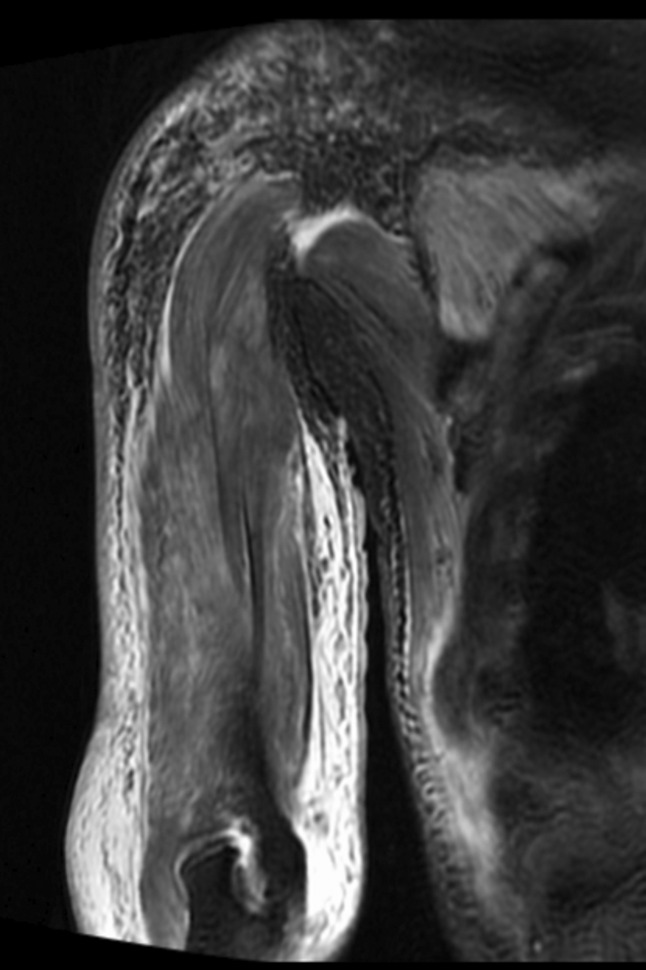
Coronal short tau inversion recovery magnetic resonance imaging reveals diffuse hyperintensity in humerus muscles.

On admission, the patient was self‐care deficit, with heliotrope rash on the face, shawl sign, macular violaceous erythema over the back, Gottron's sign on the dorsal surface of his hands, and nailfold telangiectasia (Fig. 1). Bilateral proximal lower extremities were tender, with muscle weakness in the proximal limbs. Laboratory tests revealed an elevated serum creatine phosphokinase (CPK) level (4714 U/L, normal: 57–218 U/L) but were negative for autoantibodies associated with myositis, except for anti‐TIF1γ antibody. Magnetic resonance imaging (MRI) revealed diffuse hyperintensity in the humeral muscles (Fig. 2). Skin biopsy revealed atrophic epidermis with liquefaction degeneration and melanin shedding in the basal cell layer, and sparse perivascular lymphocytic infiltration. The diagnosis was established to be TIF1γ‐positive dermatomyositis associated with durvalumab administration.

**Figure 3:**
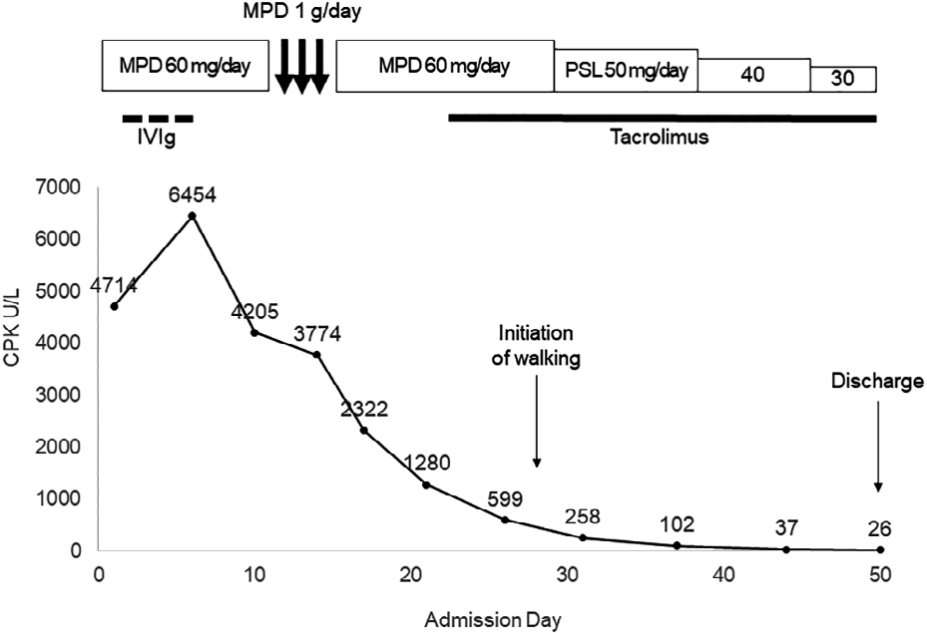
Timeline of CPK during hospitalization. CPK, creatine phosphokinase; IVIg, intravenous immunoglobulin; MPD, methylprednisolone; PSL, prednisolone.

Intravenous methylprednisolone (1 g/day for three days, then 60 mg/day) and immunoglobulin (20 g/day for five days) were initiated. Tacrolimus (3 mg/day) was added from day 23. As CPK level and muscle weakness improved, the methylprednisolone dose was reduced by 10 mg per week to 30 mg/day; subsequently, the patient was able to walk with support and was discharged on day 50 (Fig. 3).

## Discussion

This is the first report of dermatomyositis associated with durvalumab administration.

The immune mechanisms in ICI‐related dermatomyositis remains incompletely understood. A previous study on anti‐PD‐1 treatment in patients with myopathy revealed that the infiltrating cells were mainly T‐cell markers (CD3, CD4, or CD8), half of the cases had necrotic fibres, and only 69% of patients showed improvement; this suggested that ICI‐related myopathy is often refractory and early initiation of multiple immunotherapeutic agents with IVIg should be considered [2]. Tacrolimus, which inhibits the production of interleukin‐2 and upregulates T cells, may be a suitable treatment option for ICI‐induced myositis.

On the other hand, anti‐TIF1γ antibody‐positive dermatomyositis is generally associated with mild muscle disease or clinically amyopathic dermatomyositis [3]. This case developed severe muscle weakness with very high serum creatine kinase levels, which was atypical for anti‐TIF1γ antibody‐positive dermatomyositis.

Although anti‐TIF1γ antibody‐positive dermatomyositis, which often develops as a paraneoplastic process [4], could be associated with double cancers in this case, the cancers were in complete remission at the time of dermatomyositis diagnosis; hence, there is a possibility that ICIs may have played a role in developing dermatomyositis. On the Naranjo's causality assessment scale [5], the score was 4 indicating a “possible” adverse drug reaction to durvalumab.

In conclusion, durvalumab could be associated with anti‐TIF1γ antibody‐positive dermatomyositis. Continuous monitoring for muscular symptoms, CPK level, and skin rash in patients administered with durvalumab is vital.

### Disclosure Statement

Appropriate written informed consent was obtained for publication of this case report and accompanying images.

### Author Contribution Statement

Ryosuke Imai contributed to the writing of the manuscript. All authors discussed the case and commented on the manuscript.

## References

[1] Antonia SJ , Villegas A , Daniel D , et al. 2018. Overall survival with durvalumab after chemoradiotherapy in stage III NSCLC. N. Engl. J. Med. 379:2342–2350. 30280658 10.1056/NEJMoa1809697

[2] Johansen A , Christensen SJ , Scheie D , et al. 2019. Neuromuscular adverse events associated with anti‐PD‐1 monoclonal antibodies: systematic review. Neurology 92:663–674. 30850443 10.1212/WNL.0000000000007235

[3] Neil JM , and Sarah LT . 2018. Autoantibodies in myositis. Nat. Rev. Rheumatol. 14:290–302. 29674612 10.1038/nrrheum.2018.56

[4] Kaji K , Fujimoto M , Hasegawa M , et al. 2007. Identification of a novel autoantibody reactive with 155 and 140 kDa nuclear proteins in patients with dermatomyositis: an association with malignancy. Rheumatology (Oxford) 46:25–28. 16728436 10.1093/rheumatology/kel161

[5] Naranjo CA , Busto U , Sellars EM , et al. 1981. A method for estimating the probability of adverse drug reactions. Clin. Pharmacol. Ther. 30:239–245. 7249508 10.1038/clpt.1981.154

